# On the Employment of Inhalation of Ether in Some Forms of Ophthalmia

**Published:** 1848-02

**Authors:** 

**Affiliations:** Glasgow


3. *On the employment of Inhalation of Ether in some Forms of Ophthalmia.* By Dr. MACKENZIE, of Glasgow.

A recent number of the 'Annales d'Oculistique' contains a short communication from Dr. Mackenzie; and as we believe he has not published it in any English journal, we present an abstract, confirmatory as it is of the statements previously made upon the subject by MM. Cunier, Alex, and others. Having convinced himself of the power of ether in preventing the pain of operations, Dr. Mackenzie determined to examine its capability of assuaging some of the more painful affections of the eye, in which intense photophobia is a prominent symptom. "I therefore employed it in a series of cases, both in my infirmary and my private practice, and with the most satisfactory results; I have prescribed it for scrofulous ophthalmia, corneitis, sympathetic ophthalmia, neuralgia of the branches of the 5th pair, and asthenopia; and have obtained some benefit from its use in all these diseases, but principally in the first three." Three cases are given in illustration. The first of these was an example of *scrofulous ophthalmia* of three months' duration, accompanied with great photophobia. Every variety of treatment had been tried, but the temporary amendment produced was always followed by relapse at no distant period. The immediate relief afforded to the photophobia by the ether was remarkable; the patient, who had shrunk from the least access of light, being enabled to open her eyes and guide herself from a room she had been accustomed to be led out of in darkness. The amelioration continued permanent, the photophobia not returning, although the inhalation was only resorted to twice, at an interval of some days. In a case of intense *corneitis* great pain and intolerance of light prevailed, and much opacity of the cornea existed. It was one of the severest cases Dr. Mackenzie had ever seen, and yet, prompt relief of the photophobia followed the use of the ether. This was resorted to several times, until tolerance of light had become quite established, and the transparency of the cornea somewhat restored. The third case was an example of the removal, by the same agency, of a disease which usually resists our ordinary means of cure, namely *sympathetic ophthalmia* of the one eye, succeeding to a wound of the other. In this instance, the pain and intolerance were intense, and the inflammation of the various textures of the eye had not yielded to mercurialization, when great and progressive relief was attained by the inhalation. It was employed at intervals of a few days during a month.—*Annales d' Oculistique*, tom. xviii, pp. 155-9. From *British and Foreign Medical Chir. Review.*

